# Obesity and lack of breastfeeding: a perfect storm to augment risk of breast cancer?

**DOI:** 10.3389/fonc.2024.1432208

**Published:** 2024-10-25

**Authors:** Kate Ormiston, Anagh Kulkarni, Gautam Sarathy, Sara Alsammerai, Eswar Shankar, Sarmila Majumder, Kristin I. Stanford, Ramesh K. Ganju, Bhuvaneswari Ramaswamy

**Affiliations:** ^1^ Division of Medical Oncology, The Ohio State University Comprehensive Cancer Center, Columbus, OH, United States; ^2^ Dorothy M. Davis Heart and Lung Research Institute, Department of Surgery, Division of General and Gastrointestinal Surgery, The Ohio State University Wexner Medical Center, Columbus, OH, United States; ^3^ Department of Pathology, The Ohio State University Comprehensive Cancer Center, Columbus, OH, United States

**Keywords:** racial disparities, triple negative breast cancer, obesity, involution, breastfeeding

## Abstract

Triple-negative breast cancer (TNBC) is one of the most aggressive subtypes of breast cancer with higher rates of recurrence and distant metastasis, as well as decreased 5-year survival rates. Racial disparities are evident in the incidence and mortality rates of triple negative breast cancer particularly increased in young African American women. Concurrently, young African American women have multiple risk factors for TNBC including higher rates of premenopausal abdominal obesity (higher waist-hip ratio) and lower rates of breastfeeding with higher parity, implicating these factors as potentially contributors to poor outcomes. By understanding the mechanisms of how premenopausal obesity and lack of breastfeeding may be associated with increased risk of triple negative breast cancer, we can determine the best strategies for intervention and awareness to improve outcomes in TNBC.

## 1 Introduction

Breast cancer is the most commonly occurring form of cancer internationally, with more than 1 in 8 women diagnosed in their lifetime (1, 2). The chances that a woman will die of breast cancer is approximately 1 in 39, making it globally the second deadliest form of cancer in women (3). In the US, incidence rates of breast cancer diagnosis grew dramatically from the 1940s to the 1990s but have stabilized in the last two decades at approximately 130 new cases per 100,000 people (2, 4). In 2019, there were 268,000 new cases of breast cancer and 41,000 women died as a direct result of this disease (5). Predicted numbers for 2023 are similar, with 297,790 estimated new cases and 34,020 deaths (5, 6). Recent estimates suggest that nearly 4 million women with a history of breast cancer are likely currently living in the US (7).

Triple negative breast cancer (TNBC) cases generally have worse prognosis as they are characterized by aggressive growth and invasiveness compared to any other subtype of breast cancer (8, 9). This breast cancer subtype is negative for the presence of estrogen receptor (ER), progesterone receptor (PR) and human epidermal growth factor receptor 2 (HER2) (10) and do not have any targeted therapy. There are numerous risk factors for TNBC including age, age at menarche, multiparity, premenopausal obesity, lack of breastfeeding following full-term pregnancy, and duration of breastfeeding (11). TNBC is more common among women with *BRCA1* mutations (8). The African American Breast Cancer Epidemiology and Risk (AMBER) consortium utilized data collected through the Women’s Circle of Health Study, the Black Women’s Health Study, the Carolina Breast Cancer Study, and Multiethnic Cohort Study and found that the lack of breastfeeding following full-term pregnancy was associated with an increased risk of ER negative breast cancer but not ER+ breast cancer (12, 13). Additionally, this data revealed an additional risk of ER- breast cancer following each consecutive parity coupled with the absence of breastfeeding (12).

While obesity is a multifaceted, complex state that has been identified as an independent risk factor for various diseases and cancers, its relationship with breast cancer is more controversial. Several epidemiological studies have found no relationship or even a beneficial effect of higher body mass index (BMI) on breast cancer diagnosis and related outcomes (14–16). Particularly, obesity rates (BMI > 30kg/m^2^) in premenopausal breast cancer have been negatively correlated with breast cancer risk (14–16). However, several studies have shown that BMI may not be the best measurement of obesity in some populations. For instance, African American women (AAW) have higher levels of abdominal adiposity that is not accounted for in BMI measurement (17). In the East Carolina Breast Cancer Study a higher waist to hip ratio (WHR), a measurement focused on abdominal adiposity, was associated with a higher risk of TNBC in premenopausal women (18). These findings were similarly shown in the Women’s Circle of Health Study, that AAW premenopausal women with a higher WHR had a greater risk of breast cancer (19, 20). Therefore, revaluation of WHR and its association with breast cancer risk could be more appropriate in the future.

Although there have been new insights in understanding how the lack of breastfeeding impacts breast cancer risk (21), there has been no research on how obesity in combination with a lack of breastfeeding can further increase the risk of breast cancer. The lack of breastfeeding and obesity contribute to aberration in several common pathways for developing TNBC. It is important to understand how these two risk factors may interact and augment the risk. In this review, we summarize the population and biological literature on the overlapping pathways affected by lack of breastfeeding and premenopausal obesity that are associated with an increased risk of TNBC.

## 2 Breast Involution

**Figure 1:**
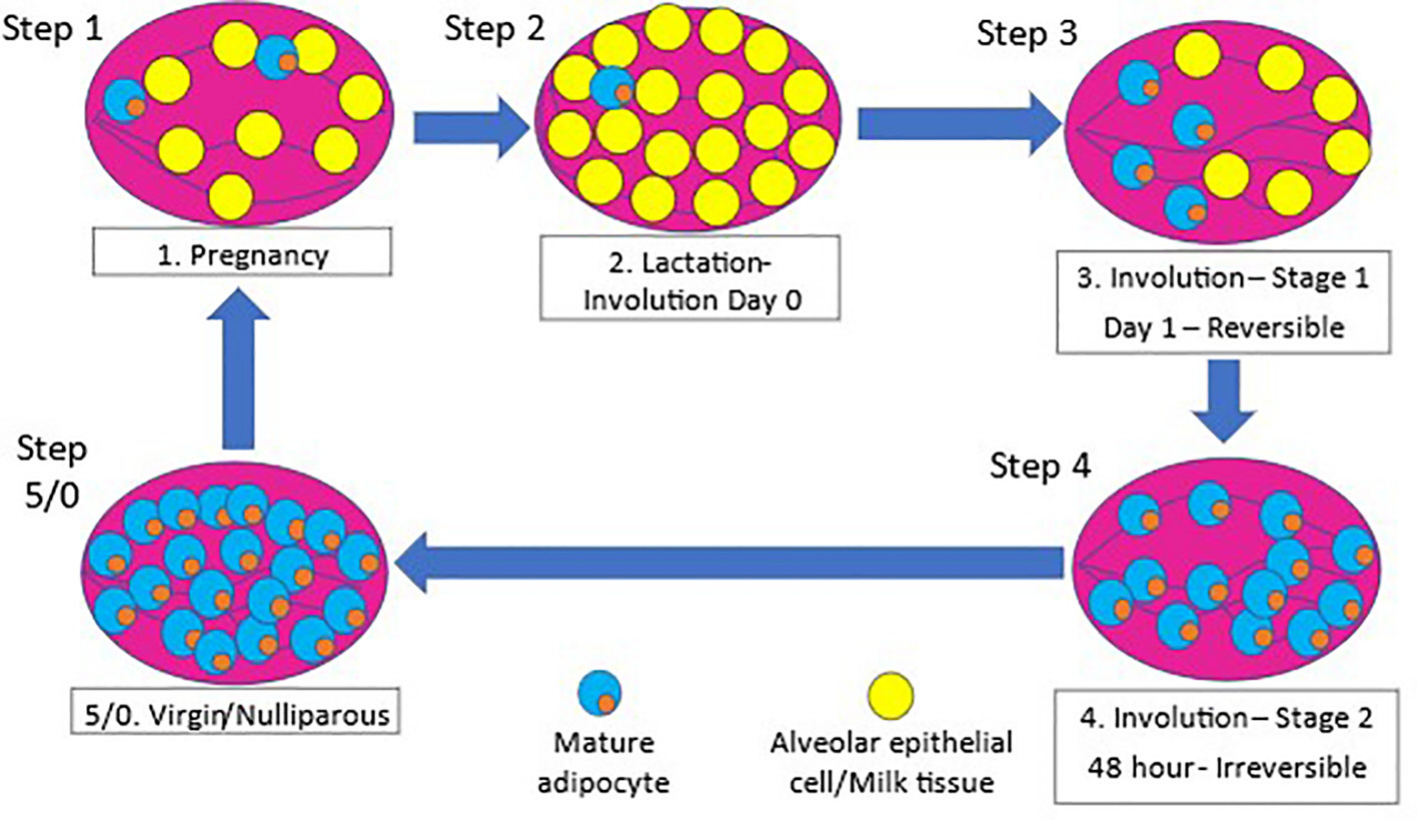
Remodeling of mammary gland during pregnancy and involution. 1) The mammary gland undergoes epithelial to alveolar differentiation during pregnancy preparing for lactation at birth, followed by massive cell death (22, 27, 29, 30). The mammary gland undergoes massive cell death and tissue remodeling as it transitions from a lactating state to a prepregnant state upon cessation of lactation after birth (22). 2) During pregnancy, the mammary gland expands dramatically through extensive epithelial cell proliferation and differentiation to alveolar cells in preparation for milk production and secretion, and this process continues throughout the period of lactation (27, 29). 3) However, upon weaning of the offspring, the gland undergoes a reversible phase of involution, where apoptotic alveolar cells shed in the lumen (27, 30). 4) This is followed by the second phase of involution within 48-72 hours of weaning, characterized by massive cell death, collapse of the alveolar structure, and adipocyte repopulation when the tissue is structurally remodeled back to its prepregnant state (27, 30).

Literature on the relationship between the lack of breastfeeding and cancer risk point towards the process of involution as being the critical window for changes increasing the risk (22). Involution of the mammary gland or breast tissue is a post-lactation process that remodels the tissue to its near pre-pregnancy state for subsequent pregnancies and lactation (23). While majority of the involution process has been elucidated using rodent models, several studies have confirmed the process of involution in humans (23–26). The process of involution is initiated by the absence of suckling and occurs in two distinct phases (27). The first phase of involution that lasts over a period of 2-3 days is reversible with re-initiation of suckling (27, 28). During this initial phase, epithelial cells undergo programmed cell death (28) and alveolar cell detachment, and dead cells accumulate in the lumen (27). Adipocytes begin to re-differentiate and re-populate the mammary gland (28). The second irreversible phase occurs over a 4-7-day period that is initiated by the breakdown of the extracellular matrix and leads to a second round of programmed cell death (27, 28). The second phase also includes collapsing of the alveoli, tissue remodeling, and adipocyte hypertrophy to a state very similar to pre-pregnancy state. The involution process is illustrated in **Figure 1**.

Through the development and use of mouse models to study this phenomenon, two distinct types of involution have been conceptualized. The term “abrupt involution” has been used to describe instances when breastfeeding is not initiated after birth or there is a short period of lactation less than 3 months (28, 31). Abrupt involution forces the mammary gland to undergo the involution and remodeling process at the peak of milk production (28, 31). On the contrary, during gradual involution when breastfeeding is prolonged greater than 6 months, alveolar cell death and remodeling of the mammary gland is more orchestrated and gradual (31). The process of gradual involution leads to remodeling of the mammary gland over a longer period of time compared to glands forced through abrupt involution (28, 31).

## 3 Overlapping link between obesity and abrupt involution on increased risk of TNBC

**Figure 2:**
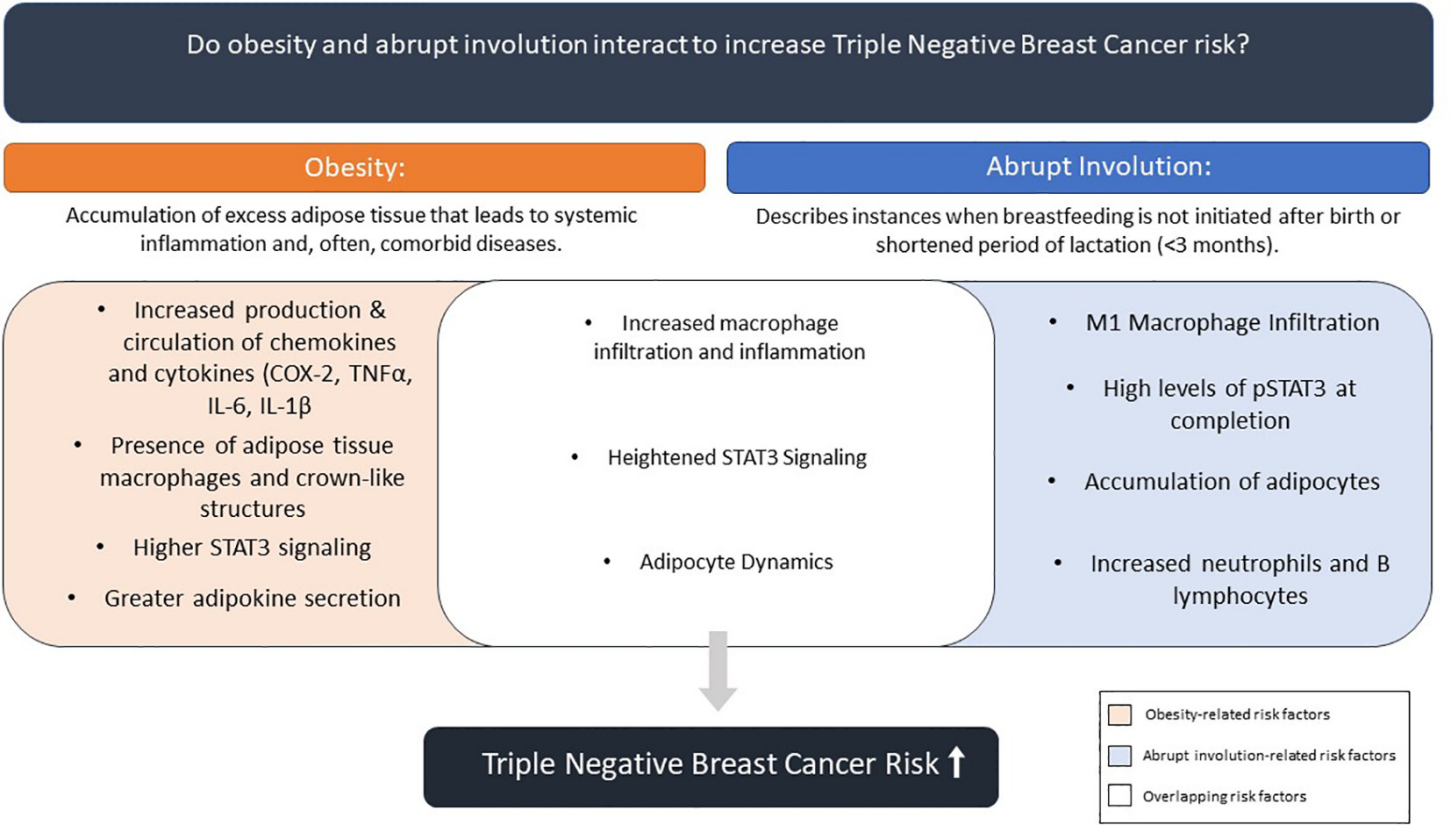
Summary of effects of obesity and abrupt involution on combined contribution to breast cancer risk.

There is overwhelming scientific evidence on role of obesity role in development of TNBC (32–37). But unlike obesity, there is limited research on abrupt involution and how lack of breastfeeding impacts the risk of developing breast cancer. Numerous epidemiological studies point to a link between lack of breastfeeding following full-term pregnancy and breast cancer risk (10–12, 21); however, majority of mechanistic studies to understand this correlation have been conducted in rodent and bovine models. Comparison of underlying mechanisms connecting the two independent TNBC risk factors, lack of breastfeeding/abrupt involution and premenopausal obesity revealed significant overlap in processes that link each factor to higher breast cancer risk (**Figure 2**). A recent study has shown that obesity induced inflammation resulted in premature involution and attributed it to zinc-mediated stress on endoplasmic reticulum (32). However, combined impacts of these two risk factors and long-term changes within the breast tissue and breast cancer risk, specifically TNBC, has yet to be studied.

### 3.1 Mammary gland Inflammation

Both obesity and abrupt involution can lead to states of increased inflammation in breast tissue (32). Obesity, on its own, is characterized as a chronic state of systemic inflammation that leads to an increase in local inflammation enhancing the risk for cancer development (34–36). Abrupt involution leads to acute inflammation in the mammary gland that sustains over time (31). However, unpublished data from our laboratory suggests abrupt involution may lead to systemic inflammation as well.

#### 3.1.1 Upregulation of cytokines and macrophage population in obesity

Multiple studies using mouse models and human tissues have shown increased inflammation, in particular macrophage infiltration, in the mammary gland of obese mice and individuals (28, 33, 35, 38). What is the link between adiposity and inflammation? In both humans and mice, as adipocytes expand in number and size, blood vessel development lags leading to a hypoxic environment (39). The lack of oxygen and over-crowding of the adipocytes causes the adipocytes to produce cytokines that increase the inflammatory state within the tissue and circulation (39). Lack of oxygen and nutrients during aberrant expansion of adipose tissue leads to adipocyte death (38, 40, 41). Infiltration of macrophages within adipose tissue, and the mammary gland, have the ability to gather around dying adipocytes (38). Increased macrophage populations may lead to a self-sustaining immune response, as macrophage infiltration leads to increased production and secretion of pro-inflammatory cytokines, such as tumor necrosis factor α (TNFα), interleukin 6 (IL-6), and interleukin 1β (IL-1β) (38). When encircling necrotic adipocytes, the macrophages form what is called a crown-like structure (CLS) (38, 40, 41). Increased inflammation, infiltration of macrophages, and formation of CLS can lead to dysregulation of adipokines, disruption of adipocyte differentiation, and changes in estrogen signaling, which are associated with increased TNBC risk (38).

Murine models of obesity have refined our understanding of cytokines in the mammary gland. In a diet-induced obesity study, C57BL/6J ovariectomized mice consuming a high-fat diet for 10 and 24 weeks showed increased levels of NFκB, IL-1β, TNFα, and COX-2 in the mammary gland compared to low-fat non-ovariectomized controls (33). This effect was further explored using a diet-induced obesity mouse model where mice were injected with E0771 cells which caused accelerated rates of TNBC development correlated with high IL-6 levels within the adipose tissue and tumor (36). Additionally, the TNBC tumors displayed increased infiltration of cancer associated adipocytes (36).

Studies have found increased CLS in the mammary gland of mice on high-fat diets (33), demonstrating an association between obesity and macrophage infiltration. In addition to an increase in the macrophage population, macrophage phenotype changes have been reported in the mammary glands of obese mice. These adipose tissue macrophages (ATM) shift to metabolically active macrophages (MMe), which are proinflammatory. MMe have been found to overexpress GPR130 ligands and produce cytokines, like IL-6, to promote stemness in cells that promote the development of TNBC. Increased concentration of MMe has been reported in breast tissues collected from obese women and was positively correlated to BMI. When cultured *in vitro*, MMe were able to activate signal transducer and activator of transcription 3 (STAT3) signaling (42), which is a pathway of interest in the development of breast cancer.

In women with TNBC, visceral adipose tissue size positively correlated with tumor size and inversely correlated with blood vessel density within the tumor (36). Additionally, pro-inflammatory cytokine IL-6 produced by the adipose tissue was higher correlated to levels within circulation and the tumor (36). In women diagnosed with breast cancer, there was a positive correlation for the presence of CLS in the breast tissue with obesity, insulin resistance, and poor prognosis (38, 40). A comparative study of breast cancer patients found that AAW had more CLS in the breast tissue than non-Hispanic black and Caucasian women (41). The macrophages surrounding adipocytes in the AAW population were shown to be highly proliferative (41).

#### 3.1.2 Upregulation of cytokines and macrophage population in abrupt involution

The inflammatory effects associated with obesity are similar to what was observed in the mammary gland following abrupt involution (31). Mouse models of involution provide evidence of an acute inflammatory response during and shortly after involution that sustains to long-term time points. For example, to understand the inflammatory process during abrupt involution of mammary gland, Stein et al. used female Balb/C mice that were forced to undergo involution on day 7 of lactation (43). During the involution process, mice displayed activation of B-cell lymphocytes and STAT3 pathways followed by increased infiltration of neutrophils and F4/80 positive macrophages in mammary gland (43). Similarly, a model using C57bl/6 and Mafia transgenic mice that forced abrupt involution at day 10 postpartum, found an increase in macrophage infiltration in wild-type mammary glands and depletion of macrophages in the transgenic mice inhibited the involution process (44). In our lab, a similar mouse model of abrupt involution was developed using FVB/n mice where forced involution was induced by removal of pups on day 7 of lactation (31). The mammary glands of these mice showed sustained activation of STAT3 pathway at day 28 postpartum and increased F4/80 macrophage infiltration even at day 56 postpartum that continued long-term to 120 days postpartum (31). In a mouse obesity model, C57bl/6 female mice on high-fat diets were found to have a higher number of macrophages in the mammary glands and this accumulation was partially responsible for induction of early involution (32).

High levels of macrophages within the mammary gland during involution has been thought to be related to upregulation of glycoprotein semaphorin A (SEMA7A) that is found on T lymphocytes (45). In C57bl/6 undergoing abrupt involution, podoplanin (PDPN), a lymphatic system marker, and marker of macrophages CD68+, were associated with SEMA7A (45). The co-expression of these gene markers is associated with increased risk and poor prognosis of breast cancer (45). This gene signature has been confirmed in breast biopsies of women undergoing involution (45). The infiltration of immune cells during abrupt involution mimics the wound healing process and promote tumor progression in D2A1 injected mice (46). Characterization of the immune environment and wound healing-like process in breast tissue was confirmed in women undergoing involution (26). Alterations of the immune environment were consistent with pro-tumorigenic conditions (26).

### 3.2 STAT3 pathway activation

STAT3 signaling pathway is an important pathway during the involution process necessary to facilitate programmed cell death. Stein et al. and Basree et al. found upregulation of STAT3 pathway activation during and after abrupt mammary gland involution (31, 43). In Balb/c mice, STAT3 mRNA levels doubled one day following forced involution and continued to be upregulated until day 3 of involution before returning towards pre-pregnancy levels (43). This early upregulation of STAT3 during involution was associated with increases in acute phase response genes such as lipopolysaccharide as well as its receptor CD14, a monocyte marker, in the luminal epithelial population (43). While STAT3 signaling was not explored during the involution process in FVB/n mice, STAT3 and phosphorylated STAT3 (pSTAT3) were found to be highly expressed in abruptly involuted mammary glands 28 days postpartum that continued to be elevated through day 56 postpartum (31). Combined, these studies suggest an early increase in STAT3 activation that helps mammary glands to return to pre-pregnancy state is re-activated when investigated after up to 120 days postpartum, demonstrating sustained inflammation.

STAT3 signaling pathway has been shown to be elevated in breast cancer, particularly TNBC (47). Cytokine IL-6 is a known transporter of STAT3 into the nucleus, showcasing a role of inflammation on the STAT3 pathway (48). In a mouse xenograft model of TNBC, blocking of STAT3 signaling was found to reduce STAT3 translocation into the nucleus, reduction of the epithelial mesenchymal transition, and promoted apoptosis of TNBC cells (48). As STAT3 has been indicated in both obesity and involution, further exploration of the combined effect of these two independent variables is warranted.

### 3.3 Adipokine alterations

In obesity, or under positive energy balance, adipocytes continuously take up free fatty acids from circulation (49). This leads to an increase in adipocyte size and crowding of adipocytes within tissues (49). As adipocytes expand, there is a decrease in oxygen availability for normal metabolic processes, which leads to hypoxia (49). Depletion or lack of oxygen available to adipose tissue has been shown to increase the development of insulin resistance and extra cellular matrix remodeling through increased secretion of leptin and decreased adiponectin gene expression (50). All these changes contribute to a pro-tumorigenic environment.

Mammary glands are composed predominantly of adipose tissue (28). In individuals who are considered overweight or obese, there is increased adiposity in the mammary glands (51). Adipose tissue secretes hormones and cytokines called adipokines (51). These hormones are a relatively new discovery with the first adipokine, leptin, being identified in the 1990s (52). While several adipokines and cytokines are released from the adipose tissue in the mammary gland, breast cancer research has focused predominantly on the effects of leptin and adiponectin, with new research emerging on the role of resistin (37, 53, 54). The role these adipokines play during and after involution has not been studied.

*Leptin:* It has been shown that pre-adipocytes and mature adipocytes secrete leptin (53). Secretion of leptin triggered by excess energy binds to the leptin receptor on the cell membrane (53) and signals the brain to reduce energy intake (55). However, overexpression of the leptin receptor and overstimulation of leptin secretion has been linked to the development of breast cancer, which could be related to higher calorie intake leading to increased adiposity (54). In addition, in postmenopausal breast cancer patients (n=42) there was increased leptin secretion compared to healthy controls (53). This increased leptin secretion was positively correlated to faster cancer progression, metastasis, and poor survival rates (53). Leptin-deficient mouse models have demonstrated reduced tumor growth rates and decrease in tumor size (56). Binding of leptin to its receptor activates multiple signaling pathways such as PI3K/AKT, MAPK/ERK1/2 and JAK/STAT, the key signaling pathways in TNBC leading to cell proliferation, migration, differentiation, anti-apoptosis, and stemness (54), connecting obesity, specifically leptin to increased tumorigenesis.

*Adiponectin:* Adiponectin is produced and secreted by adipose tissue, and an inverse correlation between adiponectin and breast cancer risk has been shown (57). It is known for its insulin-sensitizing properties, as well as regulating immune responses (56). Adiponectin was shown to prevent cell proliferation and promote apoptosis through inhibiting the AKT pathway as well as promoting increased reliance on fatty acids for energy in TNBC cell lines (57). While adiponectin can promote TNBC cell death, the role of adiponectin in breast cancer is still controversial and warrants further investigation. One study of breast cancer patients in Germany a positive correlation of adiponectin levels and increased breast cancer related mortality (58).

*Resistin:* Resistin is a relatively newer adipokine that has been investigated in connection to breast cancer risk (53). Resistin is secreted by peripheral blood mononuclear cells and macrophages in humans (53). It plays a role in the inflammatory process by targeting immune cells to increase proinflammatory cytokine production (59, 60). Studies have shown increased levels of resistin in obese humans and rodents (59). Based on the role of resistin in inflammation, a link has been proposed between resistin and insulin resistance, although overall data is inconclusive (61, 62). Resistin has been shown to increase stemness in TNBC cell populations through activation of STAT3 (59, 60). In adipocyte stem cells, resistin enhanced properties of invasion, proliferation, and mesenchymal transition when co-cultured with a TNBC cell line (60).

## 4 Discussion

Short-term breastfeeding (abrupt mammary gland involution) and obesity are highly metabolic processes that have been independently associated with increased breast cancer risk (10, 12, 21, 35, 36). In this review, we sought to provide a comparison of the mechanisms underlying these two independent risk factors, revealing numerous overlapping impacts within the breast tissue. Obesity and lack of breastfeeding following full-term pregnancy both appear lead to greater acute and chronic localized inflammation within the mammary gland related to macrophage infiltration, changes in epithelial cell population, and STAT3 activation. This supports the need to consider these two separate processes in concert and, further, whether the inflammatory effects are overlapping, additive, or synergistic.

In the US, racial disparities in mortality rates are significant after breast cancer diagnosis (8). Even with similar rates of incidence between non-Hispanic White women (NHW) and AAW, the latter face 40% higher death rates (6). This can be attributed to multiple factors, including higher poverty rates and pervasive systemic racism that undermines access to screenings and superior treatment options (6). Further incidences for high mortality rates for AAW include lack of medical coverage, barriers in accessibility to early detection and screening, more advanced stage when diagnosed, and unequal access to improvements in treatment (63).

A biological reason that contributes to the higher mortality seen in AAW is the higher incidence of the aggressive TNBC (64). While 48% of patients diagnosed with TNBC were found to have *BRCA1* mutation, the frequency of *BRCA1* mutations in AAW with TNBC is 27.9% compared to 46.2% in NHW women (65–68). AAW have multiple overlapping modifiable behavioral risk factors that contribute to increased risk of TNBC and worse outcomes, including: lack of breastfeeding, shorter duration of breastfeeding, as well as higher rates of premenopausal obesity (BMI ≥ 30 kg/m^2^) (11). Breastfeeding rates amongst AAW are far below the recommendation from pediatric health experts (69). There are many factors that lead to this disparity, such as a lack of breastfeeding education and social and familial breastfeeding support (69). The AMBER consortium was a large collaborative initiative funded by the National Cancer Institute (NCI) to understand lifestyle and genetic risk factors of breast cancer in the AAW population (13). Studies have shown that among AAW increases number of parities led to reduction of breastfeeding initiation and shorter duration of breastfeeding (70). Interestingly, the heightened risk of TNBC due to multiparity in AAW is reversed if individuals chose to breastfeed (18, 71).

While more research needs to be conducted on the lasting effects of abrupt involution, research on the combination of these two independent risk factors is imperative. By understanding the individual and combination effects of obesity and abrupt involution, intervention strategies against the detrimental effects can be developed for women who cannot or chose not to breastfeed. Research at the intersection of these two independent risk factors can impact all women who cannot or choose not to breastfeed and particularly impact AAW who have higher rates of pre-menopausal obesity, lower rates of breastfeeding, and higher incidence and mortality associated with TNBC (12). Ultimately, understanding of the combinatorial effect and development of an intervention may help to reduce racial disparities in breast cancer.

## In memoriam

We would like to dedicate this work to Dr. Bhuvaneswari Ramaswamy, who passed away after a heroic battle with breast cancer. She was incredibly passionate about her patients and research to help those with breast cancer.
